# Sigmoid Colon Intraluminal Obstruction by a Detached Recurrent Ascending Colon Liposarcoma

**DOI:** 10.7759/cureus.58065

**Published:** 2024-04-11

**Authors:** Thalia Petropoulou, Kyriacos Evangelou, Andreas Polydorou

**Affiliations:** 1 Robotic Colorectal Surgery, Aretaieio University Hospital, National and Kapodistrian University of Athens, Athens, GRC; 2 Colon and Rectal Surgery, Euroclinic Athens, Athens, GRC; 3 Faculty of Medicine, National and Kapodistrian University of Athens School of Medicine, Athens, GRC; 4 Surgery, Aretaieio University Hospital, National and Kapodistrian University of Athens School of Medicine, Athens, GRC

**Keywords:** sigmoidectomy, surgery, colorectal, generalsurgery, liposarcoma

## Abstract

Primary liposarcoma of the colon is extremely rare in the literature. We present a case of a 51-year-old male patient with recurrent ascending colon liposarcoma, which caused obstructive ileus, just a few days prior to his scheduled elective operation and led us to expedite his surgery. The procedure was scheduled to be a robotic right colectomy. After finishing the operation and extracting the specimen, the tumour could not be detected; hence, an exploratory laparotomy was performed. Findings were a large tumour in the sigmoid colon, causing complete obstruction. Sigmoidectomy was performed, in order to remove the tumour. To our knowledge, this is the first case published in the literature, reporting a colonic tumour detachment, displacement and causing distal bowel occlusion. This event highlights the importance of careful intraoperative inspection in patients with known intraluminal bowel malignancies that present with signs and symptoms of obstruction and emphasises the need for further research on the risk factors for tumour detachment and subsequent bowel occlusion.

## Introduction

Primary liposarcoma typically arises from deep soft tissues of the extremities or the retroperitoneum and is rarely encountered in the gastrointestinal tract [1]. Primary liposarcoma of the colon most frequently affects adults during the 5th to 6th decade of life, is not sex-independent and tends to recur locally [2]. 

The only effective treatment with curative intent is surgical resection [3]. Clinical manifestations include abdominal pain, rectal bleeding, weight loss, anaemia, and changes in bowel habits [4], which are also common in most types of colorectal cancer. The most common cause of mechanical bowel obstruction (MBO) in adults is bowel cancer [5]. The most common location is the sigmoid [6], in one out of five patients [7]. Pathophysiological mechanisms that lead to MBO include extraluminal intestinal compression, intraluminal occlusion or infiltration, and extensive mesenteric infiltration. Intraluminal tumours usually cause small bowel intussusception or colon lumen occlusion [8]. Therefore, MBO is a possible diagnosis in patients with symptoms of obstruction, if large bowel malignancy exists, especially for left-sided colon cancers, as the descending colon has a smaller cross-sectional area.

Bowel obstruction due to intraluminal tumours is mainly attributed to localized growth rather than distal migration and is expected to occur regionally, as infiltrated tissue confines the mass and prevents total migration. We present the case of a patient with recurrent liposarcoma of the ascending colon, who presented with MBO just a couple of days prior to his scheduled operation which was planned to be a minimally invasive right colectomy. 

## Case presentation

A 51-year-old male patient with a history of hypertension and osteoarthritis was admitted in August 2018, to a local general hospital, with abdominal distension, pain, and constipation. A CT scan was performed and large solid lesions in the right lateral portion of the peritoneal cavity were confirmed. Following laparotomy, five solid tumours from the surrounding tissues, close but not adherent to the right colon, retroperitoneal muscles and blood vessels, right ureter, and right kidney were excised. Histology reported a well-differentiated liposarcoma. The patient recovered and went home, and a regular five-year cancer follow-up was scheduled.

During the third-year follow-up (August 2021), a CT scan found an intraluminal mass, arising from the ascending colon, with similar morphological characteristics as the primary pathology and attributed to cancer recurrence. After confirming the findings with a colonoscopy and an MRI of the abdomen, an elective minimally invasive right colectomy was scheduled with en bloc resection of the surrounding tissues.

**Figure 1:**
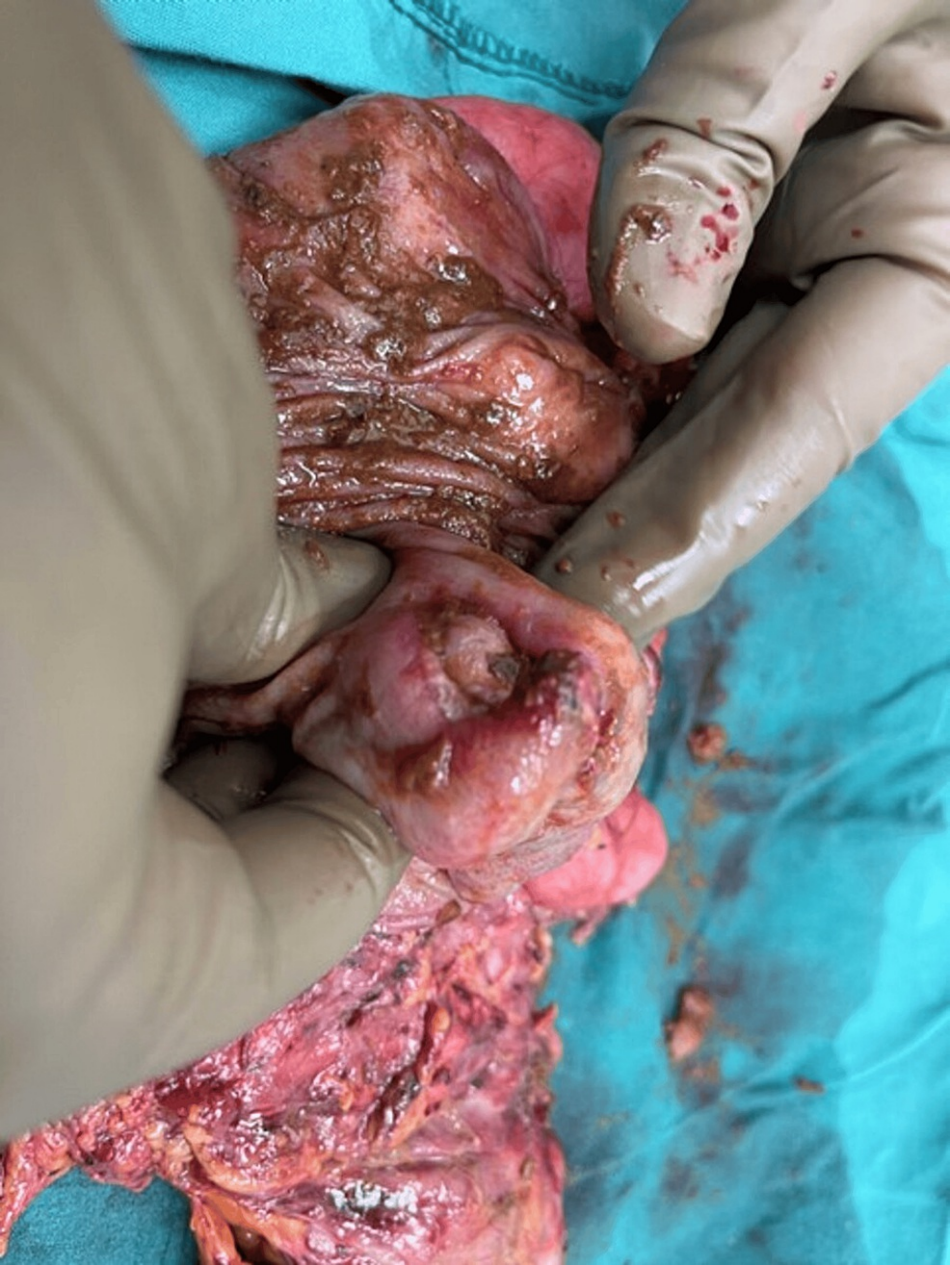
The spot inside the right colon where the detachment occurred

A few days prior to his elective operation, the patient presented to the Emergency Department of Athens Euroclinic Hospital, with abdominal pain, bloating, and bowels not opened for three days. Physical examination revealed abdominal distention, tenderness, minimal bowel sounds, absent succussion splash, and tympany on percussion. Ascending colon intraluminal obstruction by the mass was suspected and the scheduled surgery was expedited. In Figure 1, we present the spot inside the right colon where the detachment occurred.

Following surgery, the patient had an uneventful recovery. He was within our established ERAS protocol, mobilized, and ate solid food on the first postoperative day. No intravenous fluids nor opioids had been administrated from the day of the operation. There was passage of the first flatus on the second postoperative day and a first bowel motion on the third day after surgery, after which the patient was discharged home.

He had routine follow-up visits, 30 days and three months after the operation, which did not reveal any pathologies. He had his first CT scan six months after the operation, which confirmed that the patient had no recurrence. He then continued routine follow-ups with the oncology team every six months. His latest follow-up (February 2024) also did not reveal any abnormalities and the patient will continue to follow up till five years after the operation has been completed. 

**Figure 2:**
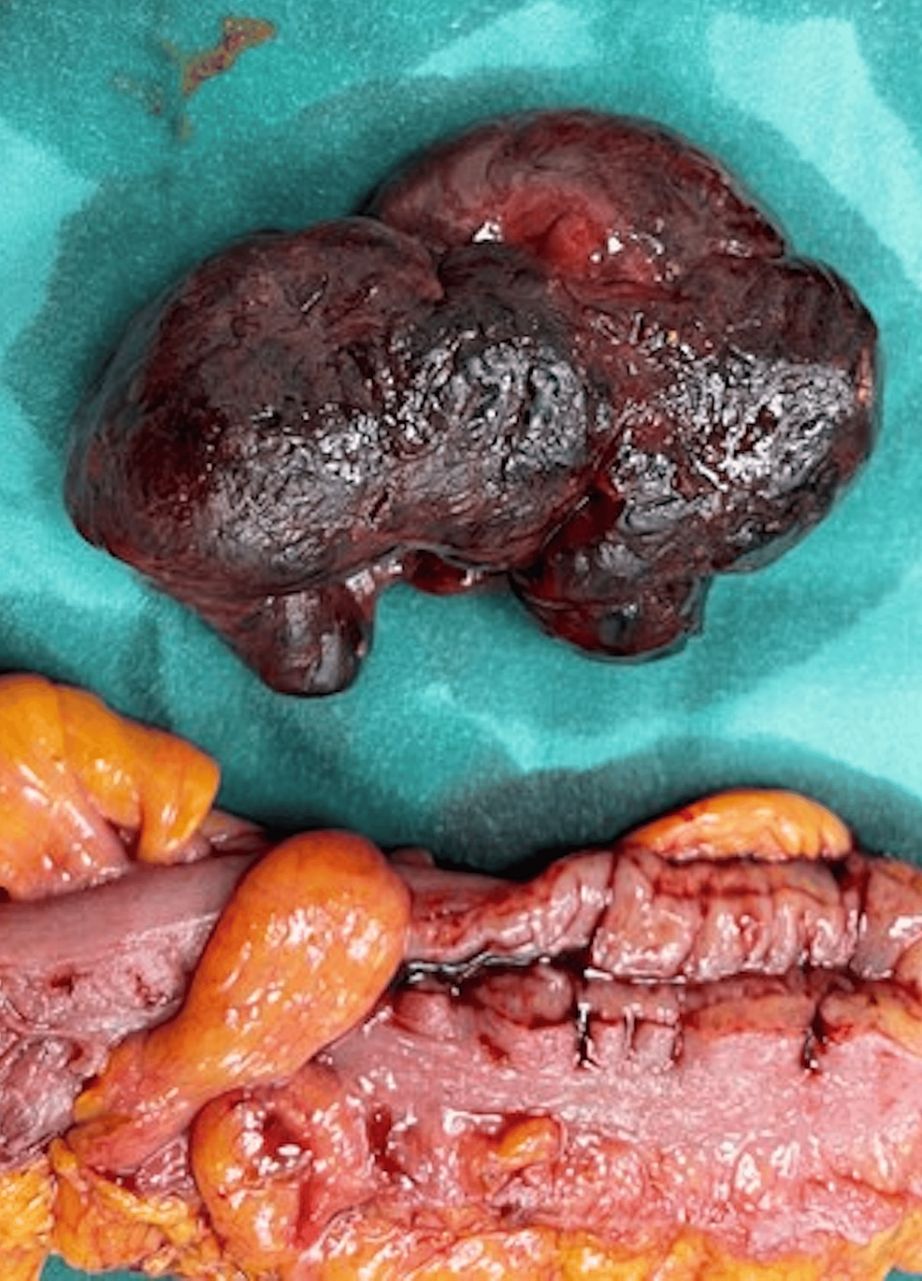
The polypoid lesion (liposarcoma) that caused the obstruction

The planned operation did not change, despite the urgency of the situation; robotic right colectomy was performed. In Figure 2, we present the polypoid lesion (liposarcoma) that caused the obstruction.

**Figure 3:**
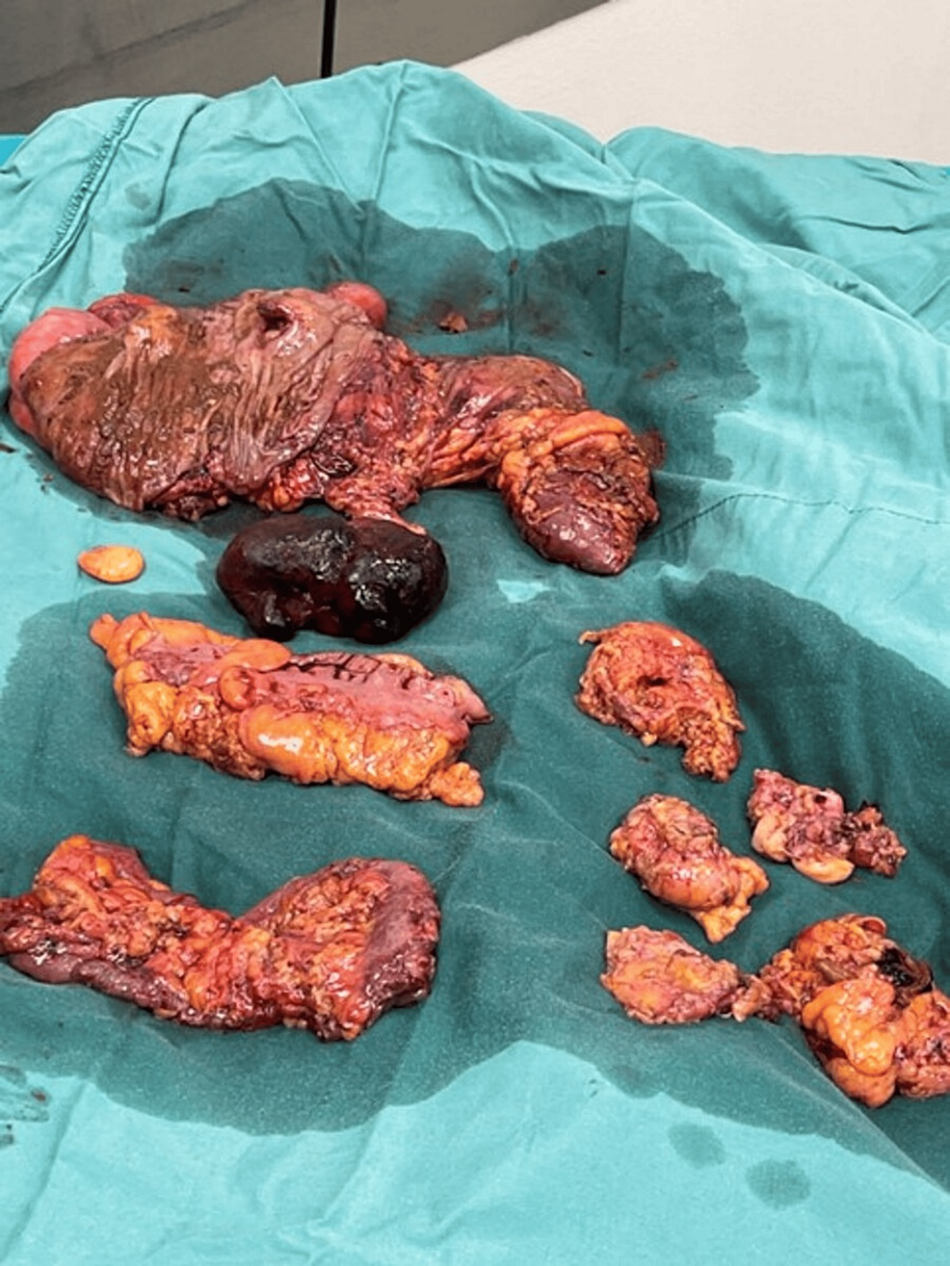
Specimen

We also removed some mesenteric nodules that were found to be irregular. Figure 3 demonstrates the exact specimens.

Investigations

**Figure 4:**
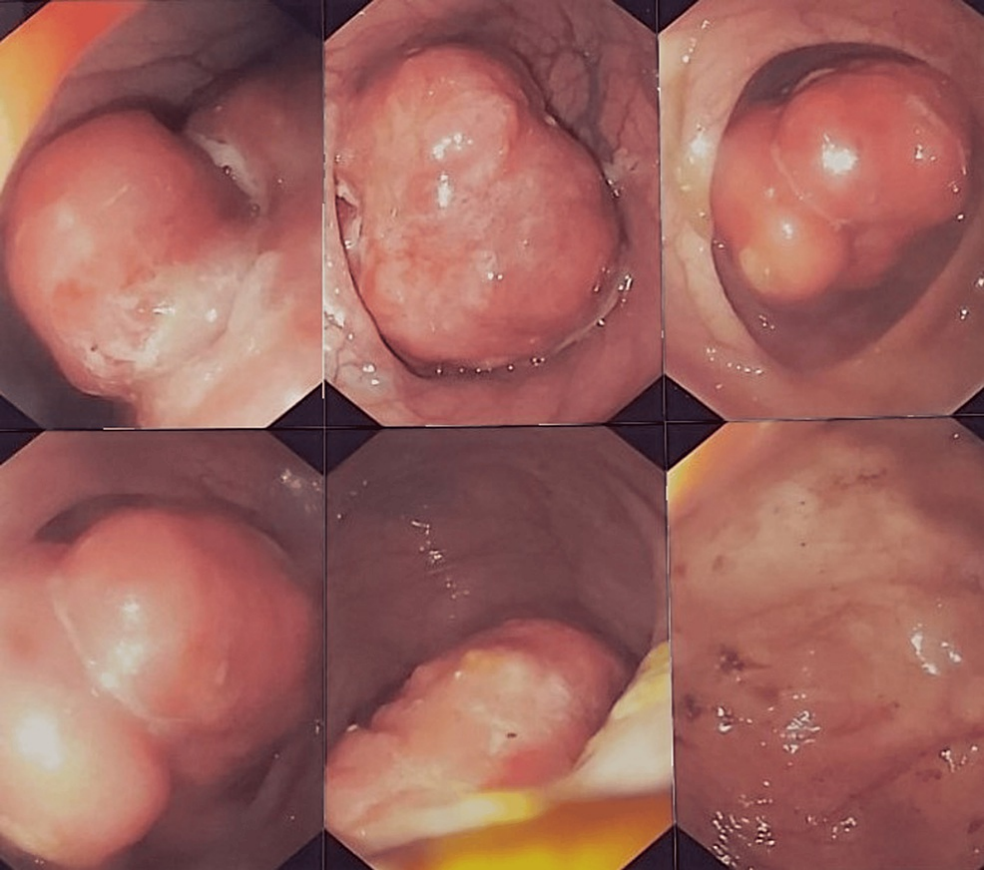
Endoscopy pictures

This new intraluminal mass was found during a colonoscopy and biopsies were taken and confirmed that this was a recurrence of the previously removed liposarcoma (Figure 4). An MRI was then also performed, for complete staging. Also, there was no differential diagnosis.

Treatment

A robotic right colectomy with our standardised technique [9] and an en bloc lesion resection of the right paracolic gutter were conducted. The en bloc resection was performed, because we saw some irregular tissue, surrounding the right colon.

**Figure 5:**
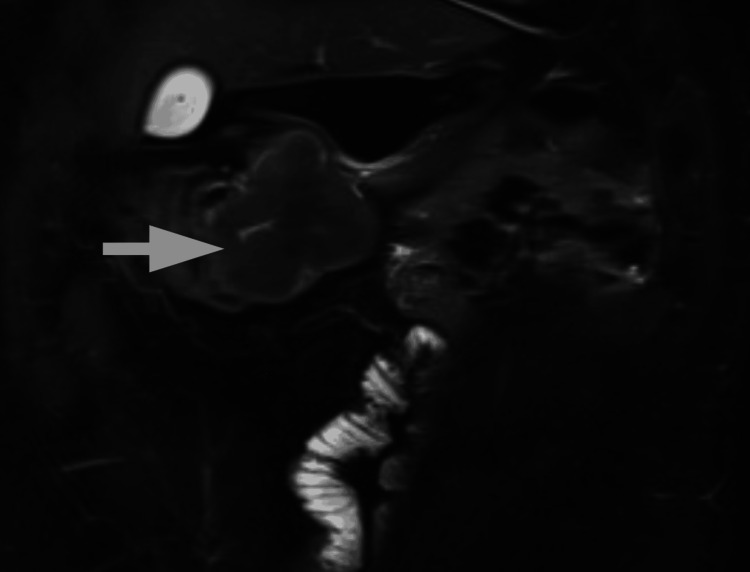
MRI scan

Following a 4-5 cm midline incision, the specimen was extracted. After palpating the resected right colon for tumour confirmation, as we do as a standard step in our technique, we could not locate the tumour. We decided to extend the incision and perform an exploratory laparotomy. We found a mass obstructing the sigmoid colon. A sigmoidectomy with an end-to-end colorectal anastomosis was performed. After removing and opening the sigmoid colon specimen, we confirmed that the initially ascending colon-confined mass had been detached and migrated to the sigmoid colon, causing mechanical obstruction (Figure 5).

## Discussion

To our knowledge, this is the first case reported in the literature that involves a malignant bowel tumour detachment and migration to a different part of the bowel, causing obstruction. Primary colon liposarcomas are very rare, with less than 30 case reports published in PubMed from January 1980 to December 2019 [10]. They mainly arise from the ascending colon, followed by the rectosigmoid and rarely the transverse colon, mesocolon and ileocaecal valve [11]. Their commonest clinical manifestations are abdominal pain and distention, but complete bowel obstruction has rarely been documented [12]. According to the most recent 2020 WHO Classification of Soft Tissue Tumours, five categories of malignant adipocytic tumours are recognized: (i) Well-differentiated liposarcoma, which includes lipoma-like, sclerosing, and inflammatory tumours, (ii) Dedifferentiated liposarcoma, (iii) Myxoid liposarcoma, (iv) Pleomorphic liposarcoma, and (v) Myxoid pleomorphic liposarcoma. Atypical lipomatous tumours are classified as intermediate-grade, locally aggressive adipocytic tumours [13].

Other types of adipocytic tumours, especially lipomas, can be detached from their pedicles and spontaneously expulsed from the rectum. It has been alleged that the risk of detachment is directly proportional to the tumour size, and videlicet larger masses are more likely to become separated from their bases and travel across the intestinal lumen [14]. So far, only benign tumour detachment and intraintestinal migration have been reported. Malignant tumours are unlikely to disengage from the intestinal wall due to their increased infiltrative potential [15]. It is much more likely for colon cancer to lead to different acute surgical complications, including haemorrhage, local obstruction, or bowel perforation [16-20]. On this basis, this case is the first ever reported involving a malignant intraluminal large bowel tumour detachment and displacement, with distal bowel occlusion. The learning point is that it is crucial to keep into consideration the possibility of mass detachment, in patients with obstruction and known proximal colonic intraluminal tumours.

In our case, if even one more similar case had been previously reported, we would have followed a different approach; specifically, a CT scan would have been performed, before we made any decisions to expedite the surgery, in order to confirm the tumour location and exclude tumour migration to the affected colon. A deeper study into the mechanical, functional, and/or biochemical factors that can contribute to malignant intestinal tumour detachment should be conducted, to shed light on potential mechanisms and avail surgeons in preventing similar events henceforward.

## Conclusions

The descending and sigmoid colons can be occluded by tumours more easily than the ascending colon, due to their smaller cross-sectional area. Although extremely rare, the event of an intraluminal ascending colon tumour detachment and migration to any peripheral section of the large bowel is feasible and can provoke an obstruction. Patients with known intraluminal colon tumours, especially larger ones, should be treated with great care in cases of obstructive symptoms; the probability of mass detachment and subsequent bowel occlusion necessitates careful investigation and appropriate interventions.
